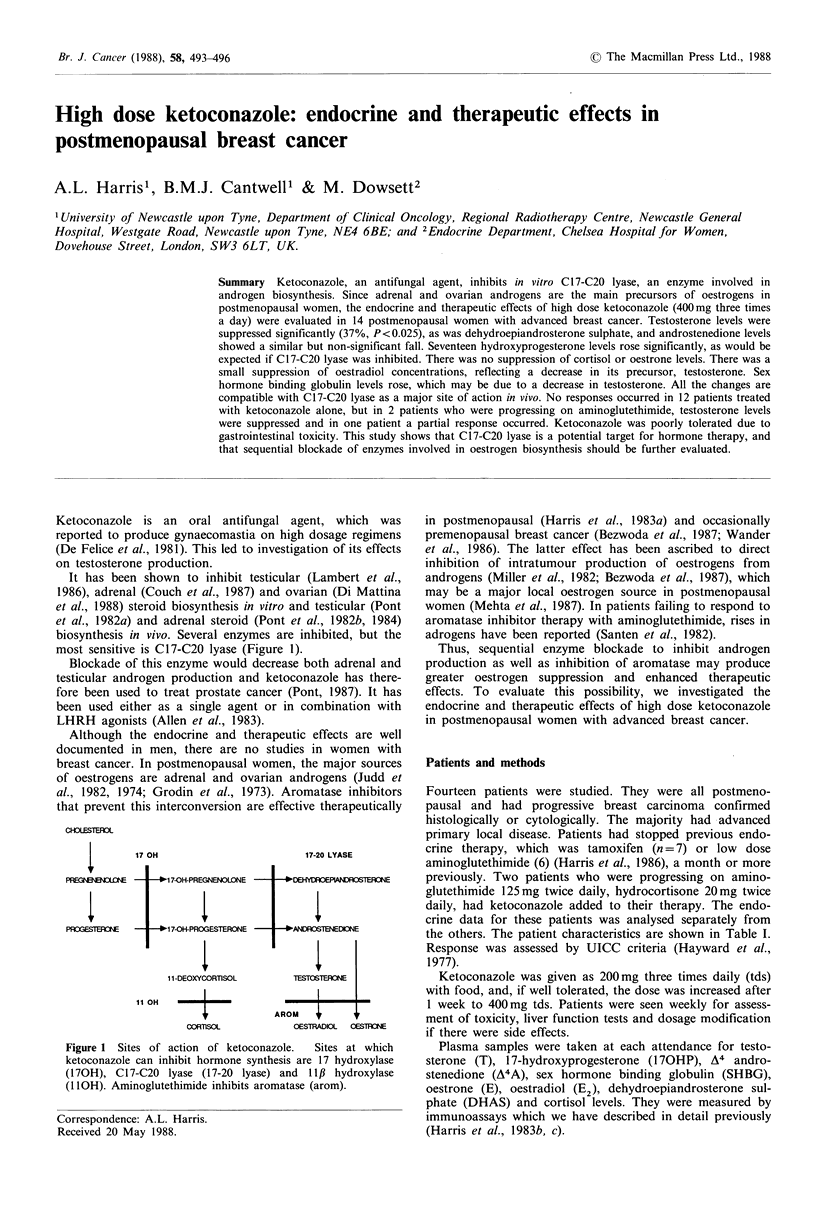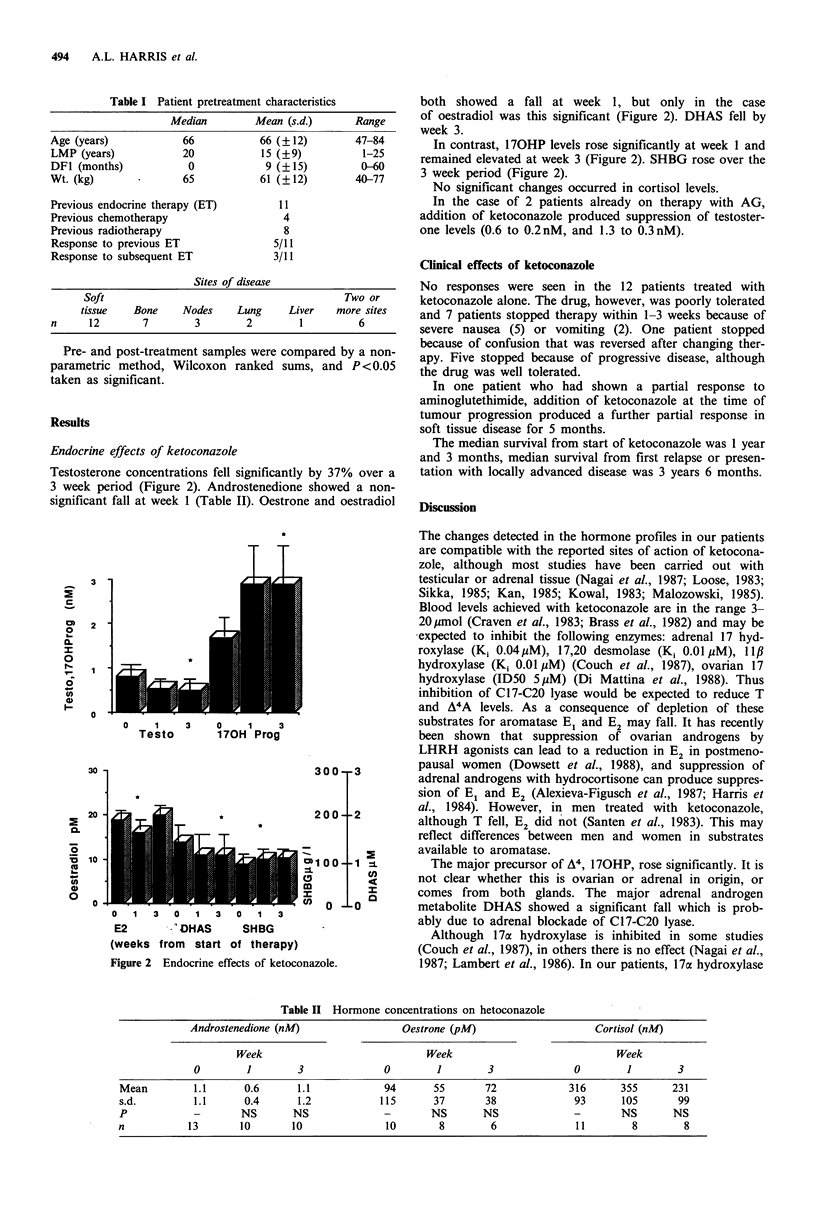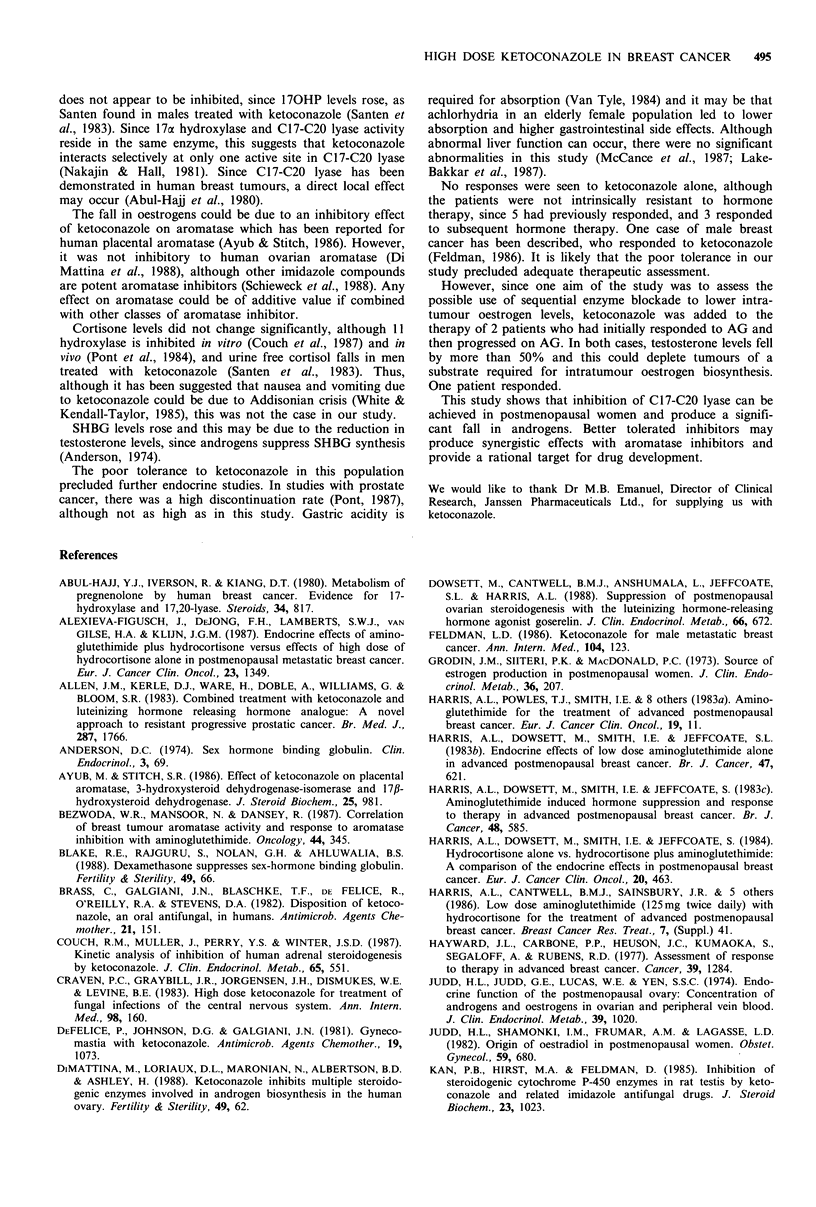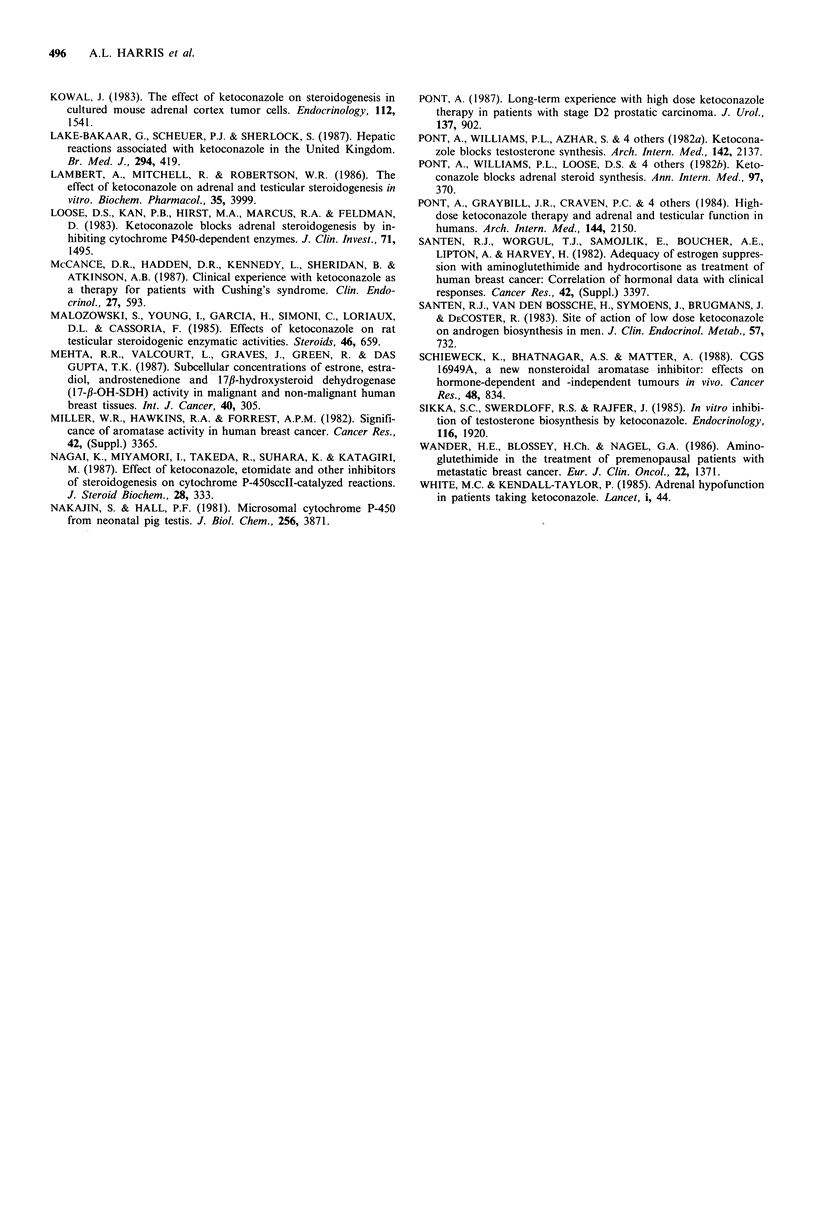# High dose ketoconazole: endocrine and therapeutic effects in postmenopausal breast cancer.

**DOI:** 10.1038/bjc.1988.247

**Published:** 1988-10

**Authors:** A. L. Harris, B. M. Cantwell, M. Dowsett

**Affiliations:** University of Newcastle upon Tyne, Department of Clinical Oncology, Newcastle General Hospital, UK.

## Abstract

Ketoconazole, an antifungal agent, inhibits in vitro C17-C20 lyase, an enzyme involved in androgen biosynthesis. Since adrenal and ovarian androgens are the main precursors of oestrogens in postmenopausal women, the endocrine and therapeutic effects of high dose ketoconazole (400 mg three times a day) were evaluated in 14 postmenopausal women with advanced breast cancer. Testosterone levels were suppressed significantly (37%, P less than 0.025), as was dehydroepiandrosterone sulphate, and androstenedione levels showed a similar but non-significant fall. Seventeen hydroxyprogesterone levels rose significantly, as would be expected if C17-C20 lyase was inhibited. There was no suppression of cortisol or oestrone levels. There was a small suppression of oestradiol concentrations, reflecting a decrease in its precursor, testosterone. Sex hormone binding globulin levels rose, which may be due to a decrease in testosterone. All the changes are compatible with C17-C20 lyase as a major site of action in vivo. No responses occurred in 12 patients treated with ketoconazole alone, but in 2 patients who were progressing on aminoglutethimide, testosterone levels were suppressed and in one patient a partial response occurred. Ketoconazole was poorly tolerated due to gastrointestinal toxicity. This study shows that C17-C20 lyase is a potential target for hormone therapy, and that sequential blockade of enzymes involved in oestrogen biosynthesis should be further evaluated.


*Br. J. Cancer* (1988), **58**, 493–496 © The Macmillan Press Ltd., 1988

### High dose ketoconazole: endocrine and therapeutic effects in postmenopausal breast cancer

A.L. Harris[1], B.M.J. Cantwell[1] & M. Dowsett[2]

[1]*University of Newcastle upon Tyne, Department of Clinical Oncology, Regional Radiotherapy Centre, Newcastle General Hospital, Westgate Road, Newcastle upon Tyne, NE4 6BE; and* [2]*Endocrine Department, Chelsea Hospital for Women, Dovehouse Street, London, SW3 6LT, UK.*

**Summary** Ketoconazole, an antifungal agent, inhibits *in vitro* C17-C20 lyase, an enzyme involved in androgen biosynthesis. Since adrenal and ovarian androgens are the main precursors of oestrogens in postmenopausal women, the endocrine and therapeutic effects of high dose ketoconazole (400 mg three times a day) were evaluated in 14 postmenopausal women with advanced breast cancer. Testosterone levels were suppressed significantly (37%, $P<0.025$), as was dehydroepiandrosterone sulphate, and androstenedione levels showed a similar but non-significant fall. Seventeen hydroxyprogesterone levels rose significantly, as would be expected if C17-C20 lyase was inhibited. There was no suppression of cortisol or oestrone levels. There was a small suppression of oestradiol concentrations, reflecting a decrease in its precursor, testosterone. Sex hormone binding globulin levels rose, which may be due to a decrease in testosterone. All the changes are compatible with C17-C20 lyase as a major site of action *in vivo*. No responses occurred in 12 patients treated with ketoconazole alone, but in 2 patients who were progressing on aminoglutethimide, testosterone levels were suppressed and in one patient a partial response occurred. Ketoconazole was poorly tolerated due to gastrointestinal toxicity. This study shows that C17-C20 lyase is a potential target for hormone therapy, and that sequential blockade of enzymes involved in oestrogen biosynthesis should be further evaluated.

Ketoconazole is an oral antifungal agent, which was reported to produce gynaecomastia on high dosage regimens (De Felice *et al.*, 1981). This led to investigation of its effects on testosterone production.

It has been shown to inhibit testicular (Lambert *et al.*, 1986), adrenal (Couch *et al.*, 1987) and ovarian (Di Mattina *et al.*, 1988) steroid biosynthesis *in vitro* and testicular (Pont *et al.*, 1982*a*) and adrenal steroid (Pont *et al.*, 1982*b*, 1984) biosynthesis *in vivo*. Several enzymes are inhibited, but the most sensitive is C17-C20 lyase (Figure 1).

Blockade of this enzyme would decrease both adrenal and testicular androgen production and ketoconazole has therefore been used to treat prostate cancer (Pont, 1987). It has been used either as a single agent or in combination with LHRH agonists (Allen *et al.*, 1983).

Although the endocrine and therapeutic effects are well documented in men, there are no studies in women with breast cancer. In postmenopausal women, the major sources of oestrogens are adrenal and ovarian androgens (Judd *et al.*, 1982, 1974; Grodin *et al.*, 1973). Aromatase inhibitors that prevent this interconversion are effective therapeutically in postmenopausal (Harris *et al.*, 1983*a*) and occasionally premenopausal breast cancer (Bezwoda *et al.*, 1987; Wander *et al.*, 1986). The latter effect has been ascribed to direct inhibition of intratumour production of oestrogens from androgens (Miller *et al.*, 1982; Bezwoda *et al.*, 1987), which may be a major local oestrogen source in postmenopausal women (Mehta *et al.*, 1987). In patients failing to respond to aromatase inhibitor therapy with aminoglutethimide, rises in adrogens have been reported (Santen *et al.*, 1982).

Thus, sequential enzyme blockade to inhibit androgen production as well as inhibition of aromatase may produce greater oestrogen suppression and enhanced therapeutic effects. To evaluate this possibility, we investigated the endocrine and therapeutic effects of high dose ketoconazole in postmenopausal women with advanced breast cancer.

**Figure 1** Sites of action of ketoconazole. Sites at which ketoconazole can inhibit hormone synthesis are 17 hydroxylase (17OH), C17-C20 lyase (17-20 lyase) and 11$\beta$ hydroxylase (11OH). Aminoglutethimide inhibits aromatase (arom).

### Patients and methods

Fourteen patients were studied. They were all postmenopausal and had progressive breast carcinoma confirmed histologically or cytologically. The majority had advanced primary local disease. Patients had stopped previous endocrine therapy, which was tamoxifen ($n=7$) or low dose aminoglutethimide (6) (Harris *et al.*, 1986), a month or more previously. Two patients who were progressing on aminoglutethimide 125 mg twice daily, hydrocortisone 20 mg twice daily, had ketoconazole added to their therapy. The endocrine data for these patients was analysed separately from the others. The patient characteristics are shown in Table I. Response was assessed by UICC criteria (Hayward *et al.*, 1977).

Ketoconazole was given as 200 mg three times daily (tds) with food, and, if well tolerated, the dose was increased after 1 week to 400 mg tds. Patients were seen weekly for assessment of toxicity, liver function tests and dosage modification if there were side effects.

Plasma samples were taken at each attendance for testosterone (T), 17-hydroxyprogesterone (17OHP), $\Delta^4$ androstenedione ($\Delta^4$A), sex hormone binding globulin (SHBG), oestrone (E), oestradiol ($E_2$), dehydroepiandrosterone sulphate (DHAS) and cortisol levels. They were measured by immunoassays which we have described in detail previously (Harris *et al.*, 1983*b*, *c*).

Correspondence: A.L. Harris.
Received 20 May 1988.

**Table I** Patient pretreatment characteristics

| | *Median* | *Mean (s.d.)* | *Range* |
|---|---|---|---|
| Age (years) | 66 | 66 (±12) | 47–84 |
| LMP (years) | 20 | 15 (±9) | 1–25 |
| DF1 (months) | 0 | 9 (±15) | 0–60 |
| Wt. (kg) | 65 | 61 (±12) | 40–77 |
| Previous endocrine therapy (ET) | | 11 | |
| Previous chemotherapy | | 4 | |
| Previous radiotherapy | | 8 | |
| Response to previous ET | | 5/11 | |
| Response to subsequent ET | | 3/11 | |

*Sites of disease*

| | *Soft tissue* | *Bone* | *Nodes* | *Lung* | *Liver* | *Two or more sites* |
|---|---|---|---|---|---|---|
| *n* | 12 | 7 | 3 | 2 | 1 | 6 |

Pre- and post-treatment samples were compared by a non-parametric method, Wilcoxon ranked sums, and $P<0.05$ taken as significant.

### Results

### *Endocrine effects of ketoconazole*

Testosterone concentrations fell significantly by 37% over a 3 week period (Figure 2). Androstenedione showed a non-significant fall at week 1 (Table II). Oestrone and oestradiol both showed a fall at week 1, but only in the case of oestradiol was this significant (Figure 2). DHAS fell by week 3.

In contrast, 17OHP levels rose significantly at week 1 and remained elevated at week 3 (Figure 2). SHBG rose over the 3 week period (Figure 2).

No significant changes occurred in cortisol levels.

In the case of 2 patients already on therapy with AG, addition of ketoconazole produced suppression of testosterone levels (0.6 to 0.2 nM, and 1.3 to 0.3 nM).

**Figure 2** Endocrine effects of ketoconazole.

### Clinical effects of ketoconazole

No responses were seen in the 12 patients treated with ketoconazole alone. The drug, however, was poorly tolerated and 7 patients stopped therapy within 1–3 weeks because of severe nausea (5) or vomiting (2). One patient stopped because of confusion that was reversed after changing therapy. Five stopped because of progressive disease, although the drug was well tolerated.

In one patient who had shown a partial response to aminoglutethimide, addition of ketoconazole at the time of tumour progression produced a further partial response in soft tissue disease for 5 months.

The median survival from start of ketoconazole was 1 year and 3 months, median survival from first relapse or presentation with locally advanced disease was 3 years 6 months.

### Discussion

The changes detected in the hormone profiles in our patients are compatible with the reported sites of action of ketoconazole, although most studies have been carried out with testicular or adrenal tissue (Nagai *et al.*, 1987; Loose, 1983; Sikka, 1985; Kan, 1985; Kowal, 1983; Malozowski, 1985). Blood levels achieved with ketoconazole are in the range 3–20 μmol (Craven *et al.*, 1983; Brass *et al.*, 1982) and may be expected to inhibit the following enzymes: adrenal 17 hydroxylase ($K_i$ 0.04 μM), 17,20 desmolase ($K_i$ 0.01 μM), 11β hydroxylase ($K_i$ 0.01 μM) (Couch *et al.*, 1987), ovarian 17 hydroxylase (ID50 5 μM) (Di Mattina *et al.*, 1988). Thus inhibition of C17-C20 lyase would be expected to reduce T and $\Delta^4$A levels. As a consequence of depletion of these substrates for aromatase $E_1$ and $E_2$ may fall. It has recently been shown that suppression of ovarian androgens by LHRH agonists can lead to a reduction in $E_2$ in postmenopausal women (Dowsett *et al.*, 1988), and suppression of adrenal androgens with hydrocortisone can produce suppression of $E_1$ and $E_2$ (Alexieva-Figusch *et al.*, 1987; Harris *et al.*, 1984). However, in men treated with ketoconazole, although T fell, $E_2$ did not (Santen *et al.*, 1983). This may reflect differences between men and women in substrates available to aromatase.

The major precursor of $\Delta^4$, 17OHP, rose significantly. It is not clear whether this is ovarian or adrenal in origin, or comes from both glands. The major adrenal androgen metabolite DHAS showed a significant fall which is probably due to adrenal blockade of C17-C20 lyase.

Although 17α hydroxylase is inhibited in some studies (Couch *et al.*, 1987), in others there is no effect (Nagai *et al.*, 1987; Lambert *et al.*, 1986). In our patients, 17α hydroxylase

**Table II** Hormone concentrations on hetoconazole

| | *Androstenedione (nM)* | | | *Oestrone (pM)* | | | *Cortisol (nM)* | | |
|---|---|---|---|---|---|---|---|---|---|
| | *Week* | | | *Week* | | | *Week* | | |
| | *0* | *1* | *3* | *0* | *1* | *3* | *0* | *1* | *3* |
| Mean | 1.1 | 0.6 | 1.1 | 94 | 55 | 72 | 316 | 355 | 231 |
| s.d. | 1.1 | 0.4 | 1.2 | 115 | 37 | 38 | 93 | 105 | 99 |
| *P* | – | NS | NS | – | NS | NS | – | NS | NS |
| *n* | 13 | 10 | 10 | 10 | 8 | 6 | 11 | 8 | 8 |

does not appear to be inhibited, since 17OHP levels rose, as Santen found in males treated with ketoconazole (Santen *et al.*, 1983). Since 17α hydroxylase and C17-C20 lyase activity reside in the same enzyme, this suggests that ketoconazole interacts selectively at only one active site in C17-C20 lyase (Nakajin & Hall, 1981). Since C17-C20 lyase has been demonstrated in human breast tumours, a direct local effect may occur (Abul-Hajj *et al.*, 1980).

The fall in oestrogens could be due to an inhibitory effect of ketoconazole on aromatase which has been reported for human placental aromatase (Ayub & Stitch, 1986). However, it was not inhibitory to human ovarian aromatase (Di Mattina *et al.*, 1988), although other imidazole compounds are potent aromatase inhibitors (Schieweck *et al.*, 1988). Any effect on aromatase could be of additive value if combined with other classes of aromatase inhibitor.

Cortisone levels did not change significantly, although 11 hydroxylase is inhibited *in vitro* (Couch *et al.*, 1987) and *in vivo* (Pont *et al.*, 1984), and urine free cortisol falls in men treated with ketoconazole (Santen *et al.*, 1983). Thus, although it has been suggested that nausea and vomiting due to ketoconazole could be due to Addisonian crisis (White & Kendall-Taylor, 1985), this was not the case in our study.

SHBG levels rose and this may be due to the reduction in testosterone levels, since androgens suppress SHBG synthesis (Anderson, 1974).

The poor tolerance to ketoconazole in this population precluded further endocrine studies. In studies with prostate cancer, there was a high discontinuation rate (Pont, 1987), although not as high as in this study. Gastric acidity is required for absorption (Van Tyle, 1984) and it may be that achlorhydria in an elderly female population led to lower absorption and higher gastrointestinal side effects. Although abnormal liver function can occur, there were no significant abnormalities in this study (McCance *et al.*, 1987; Lake-Bakkar *et al.*, 1987).

No responses were seen to ketoconazole alone, although the patients were not intrinsically resistant to hormone therapy, since 5 had previously responded, and 3 responded to subsequent hormone therapy. One case of male breast cancer has been described, who responded to ketoconazole (Feldman, 1986). It is likely that the poor tolerance in our study precluded adequate therapeutic assessment.

However, since one aim of the study was to assess the possible use of sequential enzyme blockade to lower intra-tumour oestrogen levels, ketoconazole was added to the therapy of 2 patients who had initially responded to AG and then progressed on AG. In both cases, testosterone levels fell by more than 50% and this could deplete tumours of a substrate required for intratumour oestrogen biosynthesis. One patient responded.

This study shows that inhibition of C17-C20 lyase can be achieved in postmenopausal women and produce a significant fall in androgens. Better tolerated inhibitors may produce synergistic effects with aromatase inhibitors and provide a rational target for drug development.

We would like to thank Dr M.B. Emanuel, Director of Clinical Research, Janssen Pharmaceuticals Ltd., for supplying us with ketoconazole.

### References

ABUL-HAJJ, Y.J., IVERSON, R. & KIANG, D.T. (1980). Metabolism of pregnenolone by human breast cancer. Evidence for 17-hydroxylase and 17,20-lyase. *Steroids*, **34**, 817.

ALEXIEVA-FIGUSCH, J., DEJONG, F.H., LAMBERTS, S.W.J., VAN GILSE, H.A. & KLIJN, J.G.M. (1987). Endocrine effects of aminoglutethimide plus hydrocortisone versus effects of high dose of hydrocortisone alone in postmenopausal metastatic breast cancer. *Eur. J. Cancer Clin. Oncol.*, **23**, 1349.

ALLEN, J.M., KERLE, D.J., WARE, H., DOBLE, A., WILLIAMS, G. & BLOOM, S.R. (1983). Combined treatment with ketoconazole and luteinizing hormone releasing hormone analogue: A novel approach to resistant progressive prostatic cancer. *Br. Med. J.*, **287**, 1766.

ANDERSON, D.C. (1974). Sex hormone binding globulin. *Clin. Endocrinol.*, **3**, 69.

AYUB, M. & STITCH, S.R. (1986). Effect of ketoconazole on placental aromatase, 3-hydroxysteroid dehydrogenase-isomerase and 17β-hydroxysteroid dehydrogenase. *J. Steroid Biochem.*, **25**, 981.

BEZWODA, W.R., MANSOOR, N. & DANSEY, R. (1987). Correlation of breast tumour aromatase activity and response to aromatase inhibition with aminoglutethimide. *Oncology*, **44**, 345.

BLAKE, R.E., RAJGURU, S., NOLAN, G.H. & AHLUWALIA, B.S. (1988). Dexamethasone suppresses sex-hormone binding globulin. *Fertility & Sterility*, **49**, 66.

BRASS, C., GALGIANI, J.N., BLASCHKE, T.F., DE FELICE, R., O'REILLY, R.A. & STEVENS, D.A. (1982). Disposition of ketoconazole, an oral antifungal, in humans. *Antimicrob. Agents Chemother.*, **21**, 151.

COUCH, R.M., MULLER, J., PERRY, Y.S. & WINTER, J.S.D. (1987). Kinetic analysis of inhibition of human adrenal steroidogenesis by ketoconazole. *J. Clin. Endocrinol. Metab.*, **65**, 551.

CRAVEN, P.C., GRAYBILL, J.R., JORGENSEN, J.H., DISMUKES, W.E. & LEVINE, B.E. (1983). High dose ketoconazole for treatment of fungal infections of the central nervous system. *Ann. Intern. Med.*, **98**, 160.

DEFELICE, P., JOHNSON, D.G. & GALGIANI, J.N. (1981). Gynecomastia with ketoconazole. *Antimicrob. Agents Chemother.*, **19**, 1073.

DIMATTINA, M., LORIAUX, D.L., MARONIAN, N., ALBERTSON, B.D. & ASHLEY, H. (1988). Ketoconazole inhibits multiple steroidogenic enzymes involved in androgen biosynthesis in the human ovary. *Fertility & Sterility*, **49**, 62.

DOWSETT, M., CANTWELL, B.M.J., ANSHUMALA, L., JEFFCOATE, S.L. & HARRIS, A.L. (1988). Suppression of postmenopausal ovarian steroidogenesis with the luteinizing hormone-releasing hormone agonist goserelin. *J. Clin. Endocrinol. Metab.*, **66**, 672.

FELDMAN, L.D. (1986). Ketoconazole for male metastatic breast cancer. *Ann. Intern. Med.*, **104**, 123.

GRODIN, J.M., SIITERI, P.K. & MACDONALD, P.C. (1973). Source of estrogen production in postmenopausal women. *J. Clin. Endocrinol. Metab.*, **36**, 207.

HARRIS, A.L., POWLES, T.J., SMITH, I.E. & 8 others (1983*a*). Aminoglutethimide for the treatment of advanced postmenopausal breast cancer. *Eur. J. Cancer Clin. Oncol.*, **19**, 11.

HARRIS, A.L., DOWSETT, M., SMITH, I.E. & JEFFCOATE, S.L. (1983*b*). Endocrine effects of low dose aminoglutethimide alone in advanced postmenopausal breast cancer. *Br. J. Cancer*, **47**, 621.

HARRIS, A.L., DOWSETT, M., SMITH, I.E. & JEFFCOATE, S. (1983*c*). Aminoglutethimide induced hormone suppression and response to therapy in advanced postmenopausal breast cancer. *Br. J. Cancer*, **48**, 585.

HARRIS, A.L., DOWSETT, M., SMITH, I.E. & JEFFCOATE, S. (1984). Hydrocortisone alone vs. hydrocortisone plus aminoglutethimide: A comparison of the endocrine effects in postmenopausal breast cancer. *Eur. J. Cancer Clin. Oncol.*, **20**, 463.

HARRIS, A.L., CANTWELL, B.M.J., SAINSBURY, J.R. & 5 others (1986). Low dose aminoglutethimide (125 mg twice daily) with hydrocortisone for the treatment of advanced postmenopausal breast cancer. *Breast Cancer Res. Treat.*, **7**, (Suppl.) 41.

HAYWARD, J.L., CARBONE, P.P., HEUSON, J.C., KUMAOKA, S., SEGALOFF, A. & RUBENS, R.D. (1977). Assessment of response to therapy in advanced breast cancer. *Cancer*, **39**, 1284.

JUDD, H.L., JUDD, G.E., LUCAS, W.E. & YEN, S.S.C. (1974). Endocrine function of the postmenopausal ovary: Concentration of androgens and oestrogens in ovarian and peripheral vein blood. *J. Clin. Endocrinol. Metab.*, **39**, 1020.

JUDD, H.L., SHAMONKI, I.M., FRUMAR, A.M. & LAGASSE, L.D. (1982). Origin of oestradiol in postmenopausal women. *Obstet. Gynecol.*, **59**, 680.

KAN, P.B., HIRST, M.A. & FELDMAN, D. (1985). Inhibition of steroidogenic cytochrome P-450 enzymes in rat testis by ketoconazole and related imidazole antifungal drugs. *J. Steroid Biochem.*, **23**, 1023.

KOWAL, J. (1983). The effect of ketoconazole on steroidogenesis in cultured mouse adrenal cortex tumor cells. *Endocrinology*, **112**, 1541.

LAKE-BAKAAR, G., SCHEUER, P.J. & SHERLOCK, S. (1987). Hepatic reactions associated with ketoconazole in the United Kingdom. *Br. Med. J.*, **294**, 419.

LAMBERT, A., MITCHELL, R. & ROBERTSON, W.R. (1986). The effect of ketoconazole on adrenal and testicular steroidogenesis *in vitro*. *Biochem. Pharmacol.*, **35**, 3999.

LOOSE, D.S., KAN, P.B., HIRST, M.A., MARCUS, R.A. & FELDMAN, D. (1983). Ketoconazole blocks adrenal steroidogenesis by inhibiting cytochrome P450-dependent enzymes. *J. Clin. Invest.*, **71**, 1495.

McCANCE, D.R., HADDEN, D.R., KENNEDY, L., SHERIDAN, B. & ATKINSON, A.B. (1987). Clinical experience with ketoconazole as a therapy for patients with Cushing's syndrome. *Clin. Endocrinol.*, **27**, 593.

MALOZOWSKI, S., YOUNG, I., GARCIA, H., SIMONI, C., LORIAUX, D.L. & CASSORIA, F. (1985). Effects of ketoconazole on rat testicular steroidogenic enzymatic activities. *Steroids*, **46**, 659.

MEHTA, R.R., VALCOURT, L., GRAVES, J., GREEN, R. & DAS GUPTA, T.K. (1987). Subcellular concentrations of estrone, estradiol, androstenedione and 17$\beta$-hydroxysteroid dehydrogenase (17-$\beta$-OH-SDH) activity in malignant and non-malignant human breast tissues. *Int. J. Cancer*, **40**, 305.

MILLER, W.R., HAWKINS, R.A. & FORREST, A.P.M. (1982). Significance of aromatase activity in human breast cancer. *Cancer Res.*, **42**, (Suppl.) 3365.

NAGAI, K., MIYAMORI, I., TAKEDA, R., SUHARA, K. & KATAGIRI, M. (1987). Effect of ketoconazole, etomidate and other inhibitors of steroidogenesis on cytochrome P-450sccII-catalyzed reactions. *J. Steroid Biochem.*, **28**, 333.

NAKAJIN, S. & HALL, P.F. (1981). Microsomal cytochrome P-450 from neonatal pig testis. *J. Biol. Chem.*, **256**, 3871.

PONT, A. (1987). Long-term experience with high dose ketoconazole therapy in patients with stage D2 prostatic carcinoma. *J. Urol.*, **137**, 902.

PONT, A., WILLIAMS, P.L., AZHAR, S. & 4 others (1982*a*). Ketoconazole blocks testosterone synthesis. *Arch. Intern. Med.*, **142**, 2137.

PONT, A., WILLIAMS, P.L., LOOSE, D.S. & 4 others (1982*b*). Ketoconazole blocks adrenal steroid synthesis. *Ann. Intern. Med.*, **97**, 370.

PONT, A., GRAYBILL, J.R., CRAVEN, P.C. & 4 others (1984). High-dose ketoconazole therapy and adrenal and testicular function in humans. *Arch. Intern. Med.*, **144**, 2150.

SANTEN, R.J., WORGUL, T.J., SAMOJLIK, E., BOUCHER, A.E., LIPTON, A. & HARVEY, H. (1982). Adequacy of estrogen suppression with aminoglutethimide and hydrocortisone as treatment of human breast cancer: Correlation of hormonal data with clinical responses. *Cancer Res.*, **42**, (Suppl.) 3397.

SANTEN, R.J., VAN DEN BOSSCHE, H., SYMOENS, J., BRUGMANS, J. & DECOSTER, R. (1983). Site of action of low dose ketoconazole on androgen biosynthesis in men. *J. Clin. Endocrinol. Metab.*, **57**, 732.

SCHIEWECK, K., BHATNAGAR, A.S. & MATTER, A. (1988). CGS 16949A, a new nonsteroidal aromatase inhibitor: effects on hormone-dependent and -independent tumours *in vivo*. *Cancer Res.*, **48**, 834.

SIKKA, S.C., SWERDLOFF, R.S. & RAJFER, J. (1985). *In vitro* inhibition of testosterone biosynthesis by ketoconazole. *Endocrinology*, **116**, 1920.

WANDER, H.E., BLOSSEY, H.Ch. & NAGEL, G.A. (1986). Aminoglutethimide in the treatment of premenopausal patients with metastatic breast cancer. *Eur. J. Clin. Oncol.*, **22**, 1371.

WHITE, M.C. & KENDALL-TAYLOR, P. (1985). Adrenal hypofunction in patients taking ketoconazole. *Lancet*, **i**, 44.